# Preclinical Validation of the Therapeutic Potential of Glasgow Oxygen Level Dependent (GOLD) Technology: a Theranostic for Acute Stroke

**DOI:** 10.1007/s12975-018-0679-y

**Published:** 2018-11-30

**Authors:** Graeme A. Deuchar, Josie C. van Kralingen, Lorraine M. Work, Celestine Santosh, Keith W. Muir, Chris McCabe, I. Mhairi Macrae

**Affiliations:** 1 Aurum Biosciences Ltd, 20-23 Woodside Place, Glasgow, Scotland G3 7QL UK; 2 Institute of Neuroscience & Psychology, College of Medicine, Veterinary, and Life Sciences, University of Glasgow, Glasgow, Scotland UK; 3 Institute of Cardiovascular and Medical Sciences, College of Medicine, Veterinary and Life Sciences, University of Glasgow, Glasgow, Scotland UK; 4 Institute of Neurological Sciences, Queen Elizabeth University Hospital, Glasgow, Scotland G51 4TF UK

**Keywords:** Perfluorocarbon, Stroke, MRI, Penumbra, Oxygen carrier, Oxycyte (O-PFC), Hyperoxia

## Abstract

In acute stroke patients, penumbral tissue is non-functioning but potentially salvageable within a time window of variable duration and represents target tissue for rescue. Reperfusion by thrombolysis and/or thrombectomy can rescue penumbra and improve stroke outcomes, but these treatments are currently available to a minority of patients. In addition to the utility of Glasgow Oxygen Level Dependent (GOLD) as an MRI contrast capable of detecting penumbra, its constituent perfluorocarbon (PFC) oxygen carrier, combined with normobaric hyperoxia, also represents a potential acute stroke treatment through improved oxygen delivery to penumbra. Preclinical studies were designed to test the efficacy of an intravenous oxygen carrier, the perfluorocarbon emulsion Oxycyte® (O-PFC), combined with normobaric hyperoxia (50% O_2_) in both in vitro (neuronal cell culture) and in vivo rat models of ischaemic stroke. Outcome was assessed through the quantification of lipid peroxidation and oxidative stress levels, mortality, infarct volume, neurological scoring and sensorimotor tests of functional outcome in two in vivo models of stroke. Additionally, we investigated evidence for any positive or negative interactions with the thrombolytic recombinant tissue plasminogen activator (rt-PA) following embolus-induced stroke in rats. Treatment with intravenous O-PFC + normobaric hyperoxia (50% O_2_) provided evidence of reduced infarct size and improved functional recovery. It did not exacerbate oxidative stress and showed no adverse interactions with rt-PA. The positive results and lack of adverse effects support human trials of O-PFC + 50% O_2_ normobaric hyperoxia as a potential therapeutic approach. Combined with the diagnostic data presented in the preceding paper, O-PFC and normobaric hyperoxia is a potential theranostic for acute ischaemic stroke.

## Introduction

The concept of “Time is Brain” emphasises that in general, human brain tissue becomes irreversibly damaged rapidly after arterial occlusion, with an estimated 1.9 million neurons dying every minute following occlusive stroke [1]. Following positive individual trials of intravenous recombinant tissue plasminogen activator (rt-PA) [2, 3], meta-analysis of all randomised trials of rt-PA [4] has confirmed that treatment within 4.5 h of symptom onset increased the odds of reducing disability leading to an improved ability to perform daily activities independently, with earlier treatment within this time window associated with a greater absolute benefit. An absolute increased risk of early death from intracranial haemorrhage of about 2% was outweighed by an absolute increase in disability-free survival of about 10% for patients treated within 3 h and about 5% for patients where treatment delay was between 3 and 4.5 h. More recent randomised trials of second-generation mechanical thrombectomy devices have shown improved clinical and functional outcomes, within a time window of approximately 6 h from stroke onset, in patients with anterior circulation large artery strokes, who are less successfully treated with rt-PA [5]. Most recently, benefit has been shown for both treatment with intravenous thrombolysis among patients with stroke of uncertain onset time when magnetic resonance imaging is used to define probable onset within the preceding 4.5 h, [6] and for thrombectomy up to 24 h after symptom onset when patients are selected on the basis of brain imaging findings that indicate a small volume of irreversibly damaged tissue in addition to a large artery occlusion [7, 8]. Currently a minority of patients are able to receive thrombolysis and/or thrombectomy. Access to thrombectomy in most countries requires transfer to specialist endovascular treatment centres from primary stroke centres that can initiate thrombolysis, and secondary transfer is associated with significantly longer onset-to-treatment times compared to those able to access endovascular treatment centres directly [9]. The proportion of patients with imaging patterns consistent with benefit from delayed thrombectomy is uncertain, but it is thought to represent a minority of “slow progressing” patients [10]. There is therefore considerable potential benefit for a treatment that could extend penumbral survival. Since tissue death following ischaemic stroke occurs principally because of a lack of tissue oxygen, improved oxygen delivery to penumbra could have a beneficial influence on stroke outcome.

Perfluorocarbons (PFCs) are fluorinated hydrocarbons with respiratory gas-carrying capacity superior to that of haemoglobin [11]. They are hydrophobic in nature and therefore are emulsified for intravenous use as in vivo oxygen carriers. PFCs increase tissue oxygenation through their ability to effectively decrease the plasma gap through which oxygen must travel to reach the red blood cells (RBCs) and/or vascular endothelium [12]. Because of their small particle size, PFC emulsions penetrate capillaries within ischaemic and hypoperfused microcirculation, supplying oxygen, removing carbon dioxide, reinstituting aerobic metabolism, and possibly restoring flexibility of erythrocytes stiffened by acidosis [13].

Two major mechanisms underlie the efficacy of PFCs for oxygen transport [12]. Firstly, they have a high capacity to dissolve respiratory gases including oxygen rather than binding them like a ligand. They load and unload oxygen twice as fast as haemoglobin, since gases are exchanged by simple diffusion down a partial pressure gradient. Pure PFCs can dissolve approximately 2.5 times the amount of oxygen than an equivalent volume of whole blood. Secondly, once emulsified, PFC particle size is very small (approximately 0.2 μm compared to ~ 7–8 μm for human erythrocytes), enabling better infiltration through the microcirculation and thus improving oxygen supply to tissue where perfusion of RBCs is restricted. Therefore, PFCs offer the possibility of oxygenating and thereby supporting penumbral tissue during the critical hours following stroke.

PFC’s ability to deliver oxygen to hypoxic tissue, along with the paramagnetic properties of oxygen and the different effects of oxy- and deoxyhaemoglobin on the blood oxygen level dependent (BOLD) T2* signal has been utilised to identify penumbral tissue in series of studies by our group [14–21]. These unique characteristics make PFCs potential agents with dual diagnostic [14] and therapeutic properties: providing an efficient carrier for paramagnetic oxygen and enhancing oxygen delivery to hypoxic brain tissue through any remaining plasma flow.

A number of published preclinical studies support the potential benefit of oxygenated PFCs in traumatic brain injury (TBI). Following fluid percussion injury, treatment with Oxygent (a second-generation PFC emulsion) combined with 100% inhaled oxygen for up to 4 h, improved both brain oxygen tension as well as mitochondrial oxidative activity [22]. In separate studies, Oxygent administration resulted in a significant increase in brain oxygen tension and a substantial reduction (41%) in the volume of posttraumatic brain damage [23].

The pharmacodynamic properties of Oxycyte**®** (a third-generation PFC emulsion) during haemodilution and brain injury have been investigated in rodents. Using experimental models of haemodilution, administration of intravenous Oxycyte resulted in an enhancement of tissue oxygenation at the microcirculatory level and maintained blood oxygen levels following significant blood loss [24] and effective tissue oxygenation in ischaemic states in rats and hamsters [25–27]. Oxycyte has been evaluated in rodent models of lateral fluid percussion brain injury [28], contusive spinal cord injury [29, 30] and permanent middle cerebral artery occlusion [26] where it was shown to exert a neuroprotective effect in the early phase of brain ischaemia and improve brain oxygenation. After traumatic brain injury in rats, Oxycyte improved cognitive recovery and reduced hippocampal neuronal cell loss and traumatic axonal injury [28]. Treatment with Oxycyte significantly enhanced spinal cord grey and white matter preservation following spinal cord injury as well as increasing spinal cord oxygenation [30].

Given the relatively large body of research in the literature dating back to the late 1960s, it is clear that PFCs have generated a great deal of interest for their potential use in a variety of potential indications, related to their oxygen carrying properties in vivo. Over recent years, our group has focussed on exploiting the superior oxygen carrying properties of PFCs towards the development of novel MRI-based imaging techniques to identify ischaemic penumbra following an oxygen challenge [14, 19]. Through this work, we have demonstrated the enhancement of our Glasgow Oxygen Level Dependent (GOLD) diagnostic imaging techniques to identify the metabolic penumbra in a rodent stroke model following intravenous Oxycyte (O-PFC) and hyperoxia. Regions identified as penumbra were shown to display maintained glucose metabolism. Successful translation of GOLD imaging could provide a significant advancement in the management of acute stroke patients by identifying those most likely to benefit from treatment irrespective of time from stroke onset.

With evidence of the ability to preserve penumbra from our diagnostic studies [14], we designed this program of studies to more rigorously investigate the therapeutic potential of O-PFC and hyperoxia in the acute stroke setting. These studies test the hypothesis that the administration of O-PFC in combination with an extended period of normobaric hyperoxia (50% O_2_) will exert beneficial effects on stroke outcome and oxidative stress levels in rodent stroke models.

## Material and Methods

PFC emulsion O-PFC was supplied by Tenax Therapeutics Inc. The perfluorocarbon drug substance is perfluoro(t-butylcyclohexane) (or FtBu), a saturated alicyclic perfluorocarbon, with a molecular formula of C10F20 and a molecular mass of 500.08. The finished emulsified intravenous drug product contains 60% *w*/*v* FtBu.

Animals in the control groups were administered an equivalent volume of saline.

### In Vitro Experiment

Rat neuronal cells (B50 neuroblastoma cells, European Collection of Cell Cultures, UK) were maintained in Dulbecco’s minimal essential medium supplemented with 10% (*v*/*v*) foetal bovine serum (FBS), 2 mmol/l L-glutamine, 100 U/ml penicillin and 100 μg/ml streptomycin. Twenty-four hours after plating, cells were exposed to 9 h hypoxia (1% O_2_, 5% CO_2_, balance N_2_) and serum deprivation followed by 24 h reoxygenation in complete media in either standard atmospheric air with 5% CO_2_ or hyperoxic (50% O_2_, 5% CO_2_, balance N_2_) conditions ± 10% (*v*/*v*) O-PFC. Cell lysis was then carried out.

#### Lipid Peroxidation Assay

A spectrophotometric assay (Tebu-Bio, UK) for malondialdehyde (MDA) was used to determine lipid peroxidation levels as per the manufacturer’s instructions. The MDA and hydroxyalkenal determination protocol was used and absorbance was measured at 586 nm in a spectrophotometer. All readings were normalised for protein using a BCA protein assay kit (Pierce).

### Rodent In Vivo Studies

This manuscript has been written in accordance with the ARRIVE guidelines (http://www.nc3rs.org.uk/arrive). All experiments were performed on adult male Sprague Dawley (SD) (intraluminal filament model, studies 1–3; *n* = 197, 335.1 ± 22.6 g) or Wistar (embolic stroke model, study 4; *n* = 32, 332.8 ± 21.4 g) rats obtained from Harlan, UK. Animals were randomly allocated to the study group using an online random list generator (https://www.random.org/). Investigators performing analysis to determine infarct volume were blinded to treatment allocation.

### In Vivo Rat Stroke Models

General anaesthesia was induced in all in vivo stroke studies with 5% isoflurane in nitrous oxide mixed with oxygen (70% N_2_O:30% O_2_). All surgeries were carried out under aseptic conditions. Following induction of anaesthesia, animals had oropharyngeal intubation and maintenance of anaesthesia through mechanical ventilation (approximately 3.0 ml stroke volume) with 2.5% isoflurane and N_2_O:O_2._ Prior to induction of stroke N_2_O:O_2_ was changed to medical air supplemented with ~ 5% O_2_ (26% O_2_: normoxia), delivered at a rate of approximately 60 breaths per min for all in vivo stroke surgery procedures. In animals administered hyperoxia, this was induced by increasing the balance of O_2_:medical air until a reading of 50% O_2_ was confirmed by an oxygen monitor connected to the anaesthetic outlet leading to the animal.

Body temperature was maintained at 37 °C ± 0.5 °C. A femoral vein was cannulated with polythene tubing (Portex, Smiths Medical, UK: external diameter 0.96 mm; internal diameter 0.58 mm) for administration of O-PFC or saline. Prior to recovery, the cannula was removed from the femoral vein with the small incision being repaired using diathermy.

#### Intraluminal Filament Model for Permanent and Transient MCAO

Middle cerebral artery occlusion (MCAO) was induced with an intraluminal filament. A 3.0 uncoated nylon filament with a heat-induced bulb at the tip (approximately 0.3 mm in diameter) was advanced along the left internal carotid artery for approximately 20–21 mm from the bifurcation of the external carotid artery until resistance was felt. For transient MCAO, the filament was removed following the predetermined occlusion time, the small incision in the vessel was sealed using diathermy, enabling restoration of blood flow. The animal was subsequently recovered from anaesthesia. For permanent MCAO, the filament was secured in position using ties around the internal carotid artery prior to recovery.

#### Embolic Stroke Model

##### Blood Clot Preparation

Twenty-four to 72 h prior to stroke induction, a blood clot was prepared from a donor rat. Blood was drawn from the femoral artery into a saline filled PE50 cannula, left to clot at room temperature for 2 h and stored at 4 °C for up to 72 h. When required, the clot was flushed from the cannula into sterile saline and a uniform section of ~ 30 mm was drawn into a new PE50 cannula and then pushed into a PE10 cannula, which was attached to the PE50. The clot was cut to required size within the PE10 cannula (26 ± 2 mm) for injection through the internal carotid artery.

##### Embolic Stroke Surgery

The model of embolic stroke employed was modified from a method previously described [31]. MCAO was induced through the advancement of the PE10 cannula containing the clot into the left internal carotid artery. When the tip of the cannula was approximately 1–2 mm proximal to the origin of the middle cerebral artery, the clot was injected in 75 μl saline. Rats were transferred immediately to the MRI scanner, and successful MCAO was confirmed from the initial diffusion-weighted image (DWI) scan. Heart rate and rectal temperature were continuously recorded (AcqKnowledge, Biopac Systems, CA, USA) during scanning.

### Neurological Assessment

In all studies, functional outcome following stroke was assessed with an 18-point sensorimotor assessment [32] where a high score is indicative of improved outcome (studies 1–4). In addition, the adhesive label removal test was employed to assess sensorimotor function (study 2). The time taken for the rat to both contact and remove the adhesive label from both paws was measured [33].

### Magnetic Resonance Imaging

MRI data were acquired using a Bruker Pharmascan 7 T/30 cm system with a gradient coil (internal diameter = 121 mm, 400 mT/m) and a 72 mm birdcage resonator. Following stroke surgery, anaesthetised rats were placed in the MRI rat cradle and the head was restrained using tooth and ear bars. A four-channel rat brain phased-array surface coil was placed above the rats head and secured. A pressure sensor was attached for respiratory monitoring and core body temperature was maintained at 37 ± 0.5 °C using a rectal thermocouple and a temperature controlled water jacket.

Diffusion-weighted imaging was employed to assess acute brain injury associated with embolic stroke (in vivo study 4). For quantitative determination of the apparent diffusion coefficient (ADC), a multishot spin-echo (echo planar imaging (EPI)) diffusion-weighted scan was used (TE, 22.9 ms; TR, 4000.0 ms, 2 averages, matrix, 96 × 96 mm; FOV, 25 × 25 mm^2^; 3 directions, *x*, *y*, *z*; B values, 0, 1000 s/mm^2^, 8 contiguous coronal slices of 1.5-mm thickness, 4-shot EPI). For image analysis, quantitative ADC maps (mm^2^/s) were generated for each of the eight contiguous coronal slices throughout the forebrain. Subsequent analysis of ADC maps was carried out using ImageJ software. Quantitative ADC maps in units of square millimetre per second were prepared and a reduction of 16.5% in the ADC of the mean contralateral value (excluding ventricles) was used as a threshold to determine ischaemic lesion volume [34].

T2-weighted imaging was employed to assess infarct volume 24 h (studies 3 and 4) or 7 days (study 2) after stroke using a RARE T2-weighted sequence (TE 100 ms, TR 5000 ms, rare factor 8, 2 averages, 256 × 256 matrix, 98 μm × 98 μm in-plane resolution, slice thickness 0.75 mm). The area of infarct was summed over all slices where infarcted tissue was evident. This was multiplied by slice thickness to calculate total infarct volume, which was corrected for brain swelling [35].

### H_2_O_2_ Assay

Levels of oxidative stress were measured by quantifying hydrogen peroxide (H_2_O_2_) activity present in brain tissue samples taken from the ischaemic cortex of animals selected from in vivo studies 3 and 4. An Amplex Red peroxide assay (Invitrogen, UK) was used as per manufacturer’s instructions.

### Experimental Protocols

Four in vivo stroke studies were carried out as follows: a transient intraluminal filament model of MCAO (t-MCAO) was employed for dose optimisation (study 1); efficacy assessment of O-PFC + hyperoxia was carried out in tMCAO (study 2) and in permanent MCAO (p-MCAO) (study 3); investigation of potential interactions with rt-PA was carried out in a model of embolus-induced stroke (study 4).

### Study 1—O-PFC Dose Optimisation

Under general anaesthesia, t-MCAO was induced in SD rats using the intraluminal filament model. Reperfusion was induced by subsequent removal of the filament after 90 min. 10 min prior to reperfusion, animals were randomly assigned to the following (*n* = 4–5 per group): (1) normoxia (1 ml saline i.v. plus air); (2) hyperoxia (1 ml saline i.v. plus 50% O_2_); (3) 1.5 ml/kg O-PFC i.v. + 50% O_2_; (4) 3.0 ml/kg O-PFC i.v. + 50% O_2_; (5) 4.5 ml/kg O-PFC i.v. + 50% O_2_; and (6) 6.0 ml/kg O-PFC i.v. + 50% O_2_. On recovery from anaesthesia, hyperoxia (50% O_2_) was maintained (groups 2–6) for 48 h in an oxygen tent, and thereafter, animals were returned to room air until termination (72 h). Group 1 was recovered in air. Neurological scoring was carried out at 72 h using an 18 point neurological score. The animals were then terminated with an anaesthetic overdose and infarct volume assessed on fresh coronal brain slices (6 × 1.5 mm slices) stained with TTC and corrected for brain swelling [36].

### Study 2—Efficacy of O-PFC + Hyperoxia in a t-MCAO Model

SD rats received 60 min intraluminal filament-induced MCAO. Ten minutes prior to reperfusion, animals were randomly assigned to the following (*n* = 16 per group): (1) normoxia (1 ml saline plus air); (2) hyperoxia (1 ml saline plus 50% O_2_); (3) 3.0 ml/kg O-PFC i.v. + 50% O_2_; and (4) 6.0 ml/kg O-PFC i.v. + 50% O_2_. On recovery from anaesthesia, hyperoxia (50% O_2_) was maintained (groups 2–4) for 48 h in an oxygen tent, and thereafter, animals were returned to room air until termination at day 7. Group 1 was recovered in air. Assessments of functional recovery were made at days 3 and 7 poststroke using an 18-point neurological score. Sensory and motor function was assessed at days 0, 3 and 7 using the adhesive label test [29]. When performing this test, each rat was monitored for up to a maximum of 3 min with times noted for both contact and removal of the adhesive. Final infarct size was measured at day 7 from T_2_-weighted MRI scans.

### Study 3—Efficacy of O-PFC Plus Hyperoxia in a p-MCAO Model

SD rats received intraluminal filament-induced p-MCAO with a 24 h endpoint. To inform on the minimum effective dose for clinical translation, a dose of 1.5 ml/kg O-PFC was included in this study. The therapeutic time window was also investigated by adding groups where initiation of treatment was delayed. At the relevant time point post-MCAO (indicated below), animals were randomly assigned to one of the following groups (*n* = 14 per group): (1) normoxia (saline i.v. + air starting 60 min post-MCAO); (2) hyperoxia (saline i.v. + 50% O_2_) starting 60 min post-MCAO; (3) 1.5 ml/kg O-PFC i.v. + 50% O_2_ starting 60 min post-MCAO; (4) 3 ml/kg O-PFC i.v. + 50% O_2_ starting 60 min post-MCAO; (5) hyperoxia (saline i.v. + 50% O_2_) starting 3 h post-MCAO; (6) 1.5 ml/kg O-PFC i.v. + 50% O_2_ starting 3 h post-MCAO; and (7) 3 ml/kg O-PFC i.v. + 50% O_2_ starting 3 h post-MCAO. On recovery from anaesthesia, hyperoxia (50% O_2_) was maintained (groups 2–7) for 24 h in an oxygen tent. Group 1 was recovered in air. An 18-point neurological score [32] was carried out at 24 h poststroke followed by infarct volume measurement from T_2_-weighted MRI scans. The animal was subsequently terminated and the brain was removed. The ipsilateral hemisphere was dissected free, and the cortical tissue was separated. This tissue was snap frozen in liquid nitrogen and stored at − 80 °C for analysis of oxidative stress. This was done using an Amplex® Red Hydrogen Peroxide/Peroxidase Assay to measure levels of hydrogen peroxide in the ischaemic cortex of brain tissue from animals (*n* = 5) selected at random from each group.

### Study 4—Investigation of Safety of O-PFC and Hyperoxia in Combination with rt-PA in an Embolic Stroke Model

#### Embolic Stroke Model—Blood Clot Preparation

Adult male Wistar rats were used for this study. The preparation of the blood clot and the embolic stroke surgery was performed as described above with the femoral vein also being cannulated for administration of O-PFC and rt-PA.

#### Treatment

Treatment was initiated at approximately 75 min following introduction of blood clot and baseline DWI scanning to confirm evidence of early ischaemic damage (6 animals with no hyperintensity on DWI scans were excluded from the study). Animals were randomly assigned to one of the following groups (*n* = 8 per group): (1) 3 ml/kg saline + normoxia; (2) 3 ml/kg O-PFC + hyperoxia (50% O_2_); (3) 0.9 ml/kg rt-PA + normoxia; (4) 0.9 ml/kg rt-PA + 3 ml/kg O-PFC + hyperoxia (50% O_2_). In groups receiving rt-PA, the first 10% of the dose was given as a bolus with the remaining dose infused over 45 min. In group 4, treatment with O-PFC and hyperoxia was commenced immediately prior to rt-PA. At the end of the scanning protocol, animals were returned to the operating theatre for removal of the femoral cannula and recovery from anaesthesia. In groups 2 and 4 where treatment included hyperoxia, this was maintained for 24 h in an oxygen tent. Groups 1 and 3 were recovered in air. An 18-point neurological score [32] was carried out at 24 h poststroke followed by infarct volume measurement from T_2_ MRI scans. The animal was subsequently terminated and the brain was removed. The ipsilateral hemisphere was dissected free, and the cortex was separated and snap frozen in liquid nitrogen and stored at − 80 °C for analysis of oxidative stress using the Amplex® Red Hydrogen Peroxide/Peroxidase Assay to measure levels of hydrogen peroxide in the ischaemic cortex of brain tissue from animals (*n* = 5) selected at random from each group.

### Statistics

In vitro experiments were performed in triplicate on ⩾ 3 independent occasions and analysed by unpaired Student’s *t* test. Comparison of final infarct size and label contact and removal time between in vivo groups was analysed using one-way analysis of variance (ANOVA) with Bonferroni’s posttests for multiple comparisons. Comparison of 18-point neurological score data between in vivo groups was analysed using a Kruskall–Wallis non-parametric test with Dunn’s posttests for multiple comparisons. Comparison of changes in lesion size (study 4) over time and between treatment groups was analysed using a repeated measures (mixed model) ANOVA with Bonferroni’s posttests. Data in figures and tables are presented as mean ± SD.

## Results

### In Vitro Model of Hypoxia and Reoxygenation

**Fig. 1:**
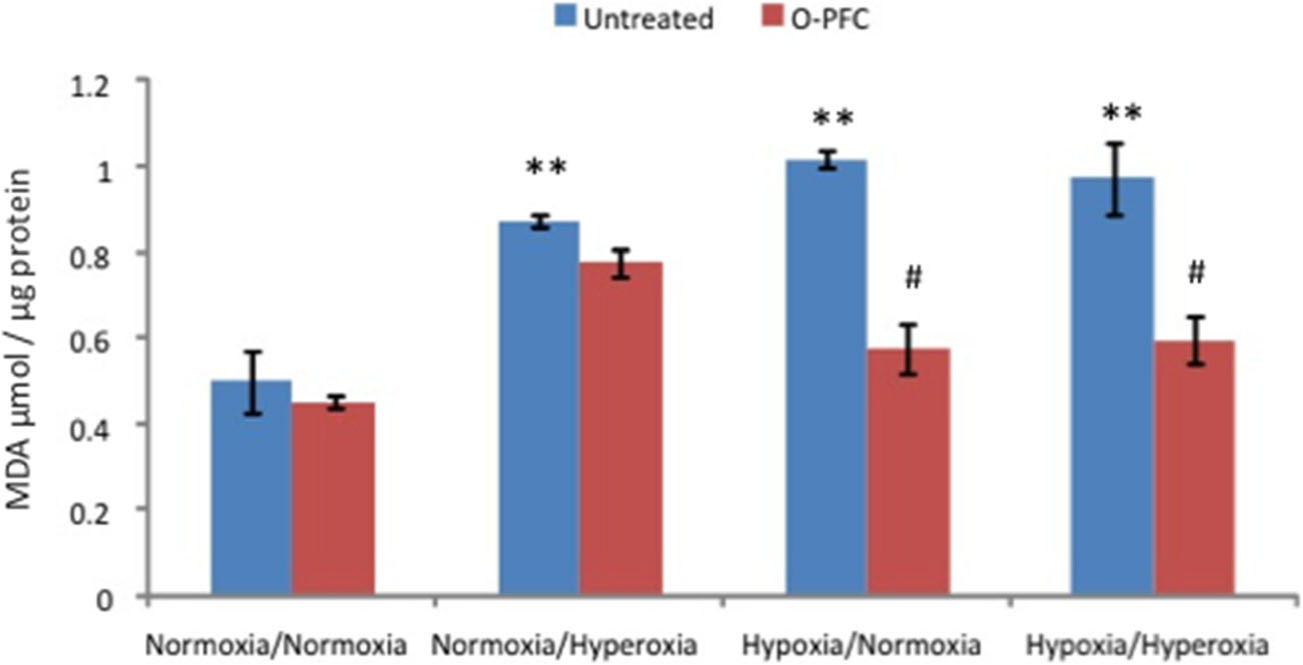
The effect of 9-h normoxia or hypoxia (1%O_2_:5%CO_2_:balanceN_2_) followed by 24 h of normoxia or hyperoxia (50%O_2_:5%CO_2_:balanceN_2_) on MDA levels in rat B50 neuroblastoma cells in the presence (red bars) or absence (blue bars) of O-PFC (10% *v*/*v*). Results shown are representative of an experiment performed on three separate occasions (*n* = 3). Data are presented as mean ± SEM. **p < 0.01 vs untreated normoxia/normoxia; #*p* < 0.01 vs untreated hypoxia/normoxia or untreated hypoxia/hyperoxia

In vitro studies in a neuronal cell line showed that, as expected, MDA levels increased after exposure to hypoxia. Treatment with O-PFC (10% *v*/*v*) during reoxygenation significantly reduced levels of lipid peroxidation irrespective of whether reoxygenation was delivered with normoxic or hyperoxic (50%) O_2_ levels (Fig. 1).

### In Vivo Study 1—Dose Optimisation

**Fig. 2:**
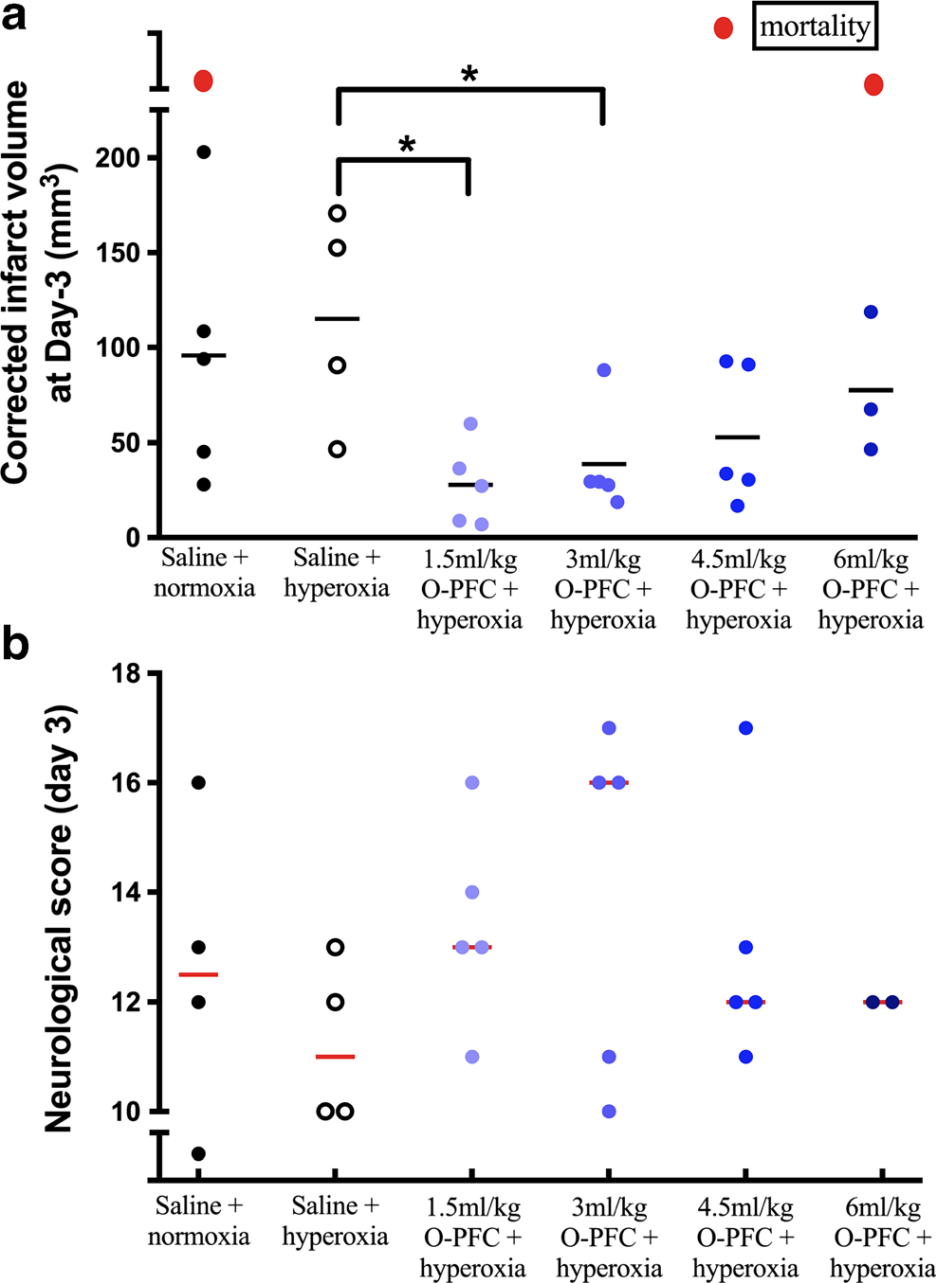
Study 1, dose optimisation study in transient (90 min) MCAO model (*n* = 4–5 per group). **a** Infarct volume 3 days after stroke. Red symbols represent animals that died during the experiment. **b** Eighteen-point neurological score 3 days after stroke. *p < 0.05, 1.5 ml/kg and 3.0 ml/kg O-PFC + hyperoxia vs saline + hyperoxia . One-way ANOVA with Bonferroni correction for multiple comparisons. High score = improved function, red bars indicate median

This study demonstrated no reduction in infarct size (Fig. 2a) using hyperoxia alone compared to the normoxia control (normoxia, mean lesion size = 95.7 ± 68.6 mm^3^*n* = 5; hyperoxia, mean lesion size = 115.0 ± 57.1 mm^3^, *n* = 4). Infarct volumes were smaller in the 1.5 and 3 ml/kg O-PFC + hyperoxia groups (27.8 ± 21.9 mm^3^ and 38.6 ± 28.0 mm^3^, respectively, reaching statistical significance, *p* < 0.05) when compared to the saline + hyperoxia group (115 ± 57.1 mm^3^) but not the saline + normoxia group (95.6 ± 68.6 mm^3^). However, there was a potential loss of efficacy in groups receiving higher (4.5 and 6 ml/kg) doses of O-PFC + hyperoxia, with mean infarct volumes of 52.8 ± 36.1 mm^3^ and 77.5 ± 37.1 mm^3^, respectively (Fig. 2a). One animal died in the saline normoxia group and one in the 6 ml/kg O-PFC + hyperoxia group. The 18-point neurological score (Fig. 2b) displayed a trend towards functional improvements with the lower doses (1.5 and 3 mg/kg O-PFC + 50% O_2_). Consequently, 3 ml/kg O-PFC + 50% O_2_ was chosen as the dose for the larger, more detailed efficacy study using the t-MCAO (60 min) model, with the 6 ml/kg dose included to investigate the possible loss of efficacy.

### In Vivo Study 2—Efficacy of O-PFC in a Transient MCAO Model

**Fig. 3:**
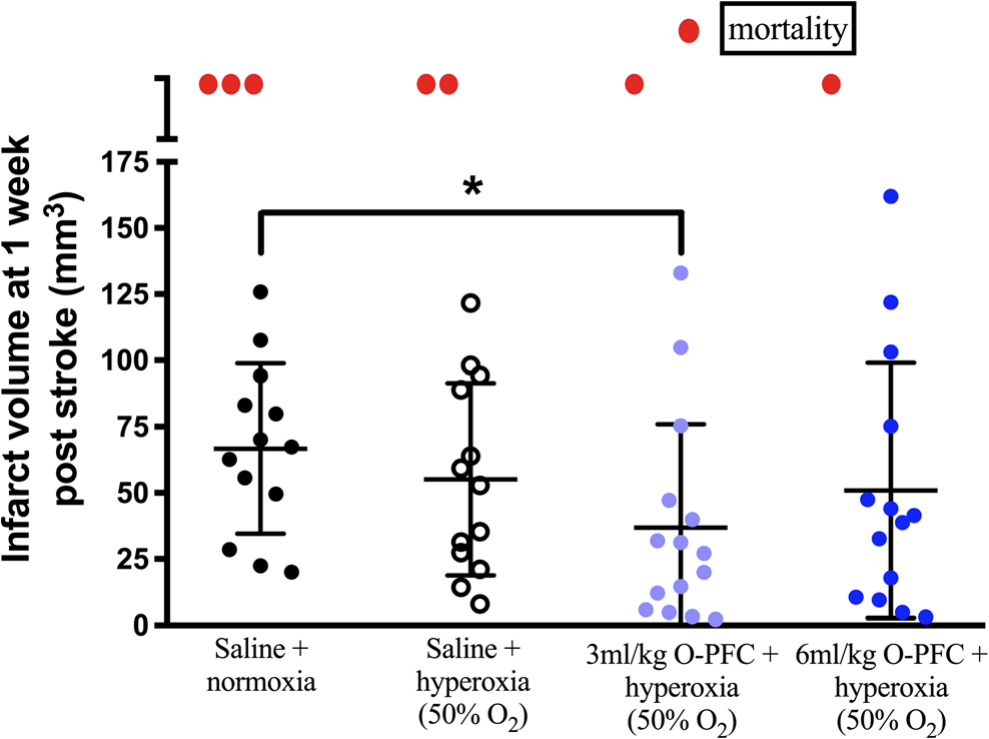
Study 2. Infarct volume was measured at 7 days poststroke (60 min transient MCAO). Data presented as mean ± SD (*n* = 16 per group). Red symbols represent animals that died during the experiment. *p < 0.05 vs saline + normoxia. One-way ANOVA with Bonferroni’s multiple comparison test

As illustrated in Fig. 3, infarct size 7 days after t-MCAO was significantly smaller in the 3 ml/kg O-PFC + hyperoxia group compared to the normoxic controls (mean lesion size = 36.9 ± 39.0 mm^3^ compared to normoxic mean lesion size = 66.7 ± 32.2 mm^3^, *p* < 0.05, Fig. 3), but not with the higher (6 ml/kg) dose of O-PFC + hyperoxia. Mortality was higher in the saline-treated groups (19% in saline + normoxia; 12.5% in saline + hyperoxia) compared to the O-PFC-treated groups (6% in both groups).

**Fig. 4:**
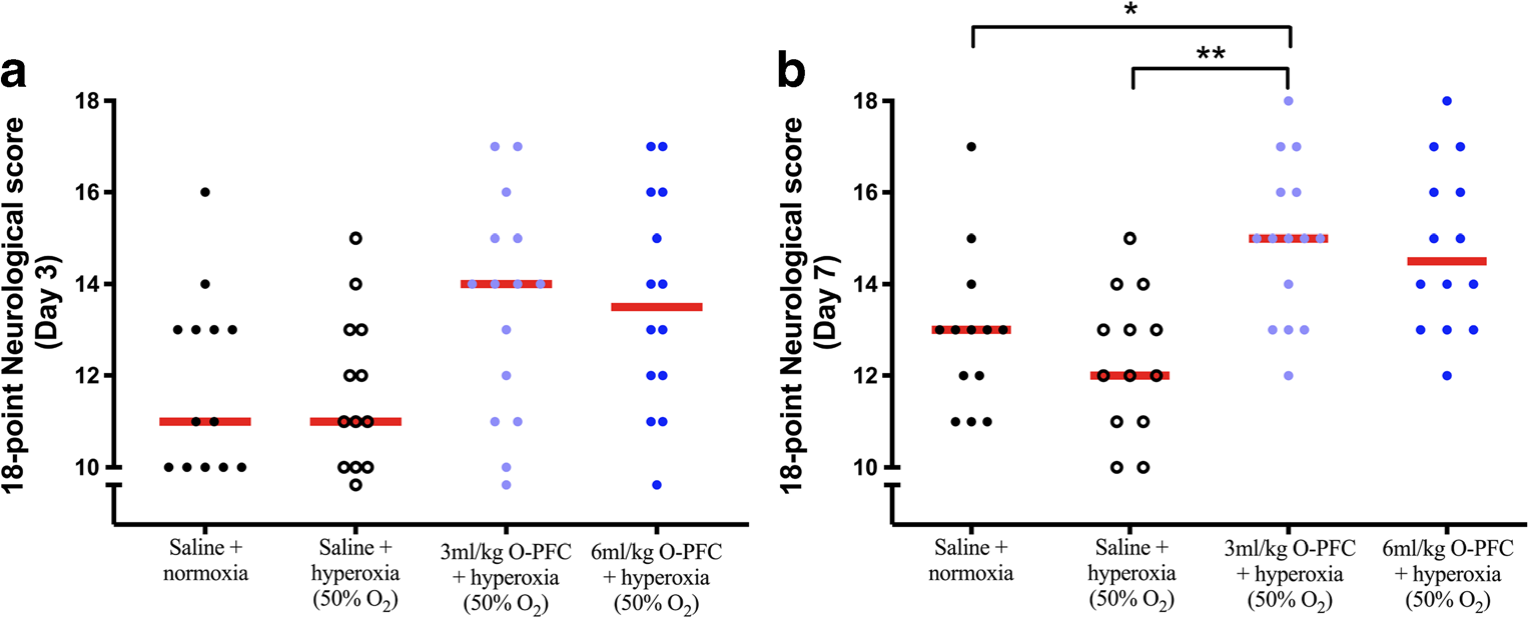
Study 2. Day 3 (**a**) and day 7 (**b**) data for the 18-point neurological score following 60 min tMCAO. Red bars indicate median (*n* = 16 per group). *p < 0.05, 3 ml/kg O-PFC + hyperoxia vs saline + normoxia; **p < 0.01, 3 ml/kg O-PFC + hyperoxia vs saline + hyperoxia, Kruskall–Wallis non-parametric test with Dunn’s post hoc multiple comparison. High score = improved function, red bars indicate median

**Fig. 5:**
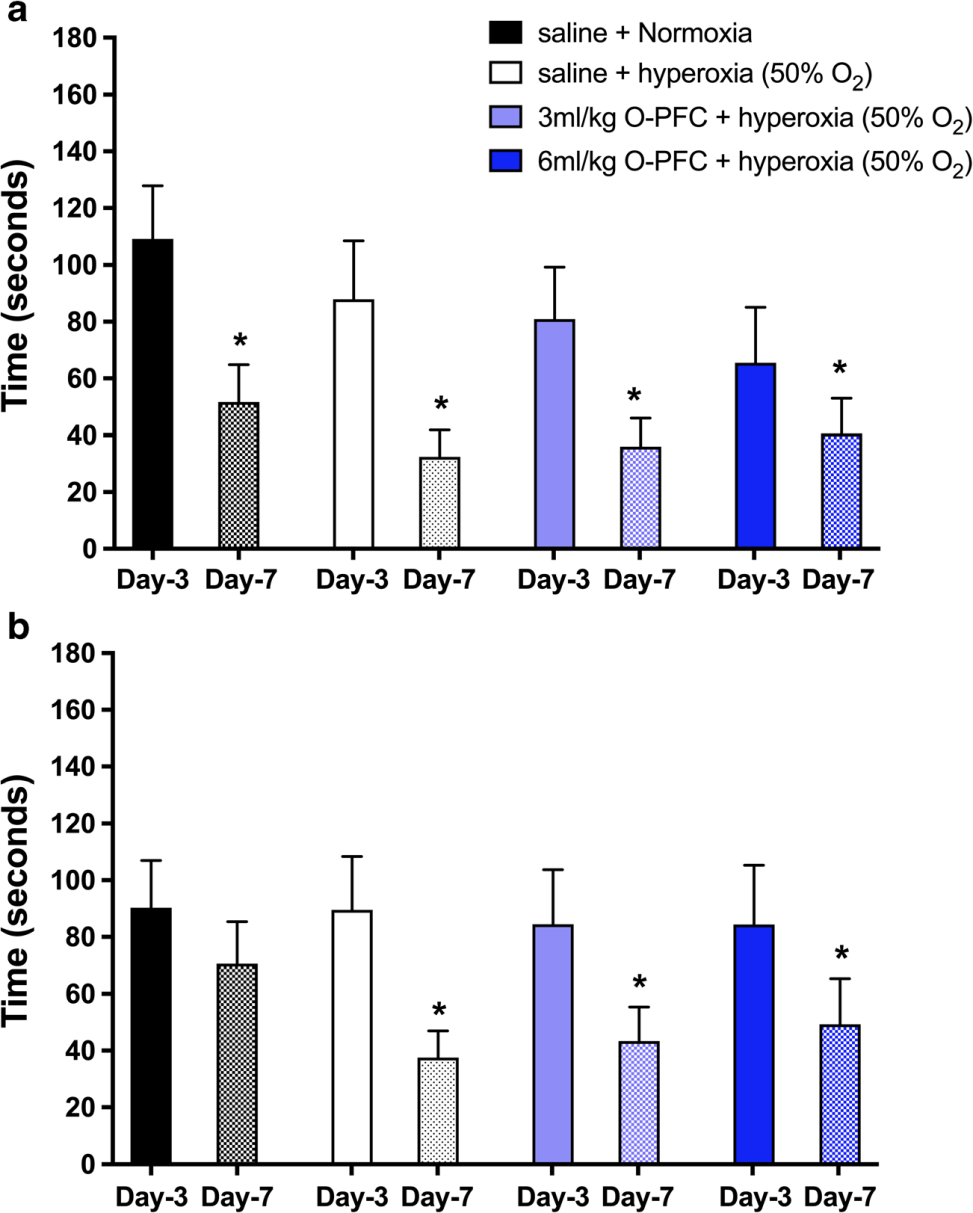
Study 2. Adhesive label test. **a** Lag time between the ipsilateral and contralateral paw to contact the adhesive label at day 3 and 7 poststroke. **b** Lag time between ipsilateral and contralateral paw to remove the adhesive label at days 3 and 7 poststroke. Data are presented as mean ± SD. Normoxia (*n* = 13); hyperoxia (50% O_2_) (*n* = 13); 3 ml/kg O-PFC + 50% O_2_ (*n* = 15); 6 ml/kg O-PFC + 50% O_2_ (*n* = 14). *p < 0.05 indicates significant improvement in the lag time between day 3 and day 7 (paired *t* test)

There was also evidence of improved functional outcome in the 3 ml/kg O-PFC + hyperoxia-treated group by day 7 (Fig. 4). The adhesive label test, used to asess sensorimotor function, revealed no significant difference in preference to contact and remove labels from left and right forepaws at day 0 (prestroke). By day 3 poststroke, there was a significant impairment in the ability to contact and remove the label contralateral to stroke in all groups. Data is shown for the lag time between the ipsilateral and contralateral paw in terms on time to contact (Fig. 5a) and time to remove (Fig. 5b) the adhesive label. No difference was observed between the groups on either day 3 or day 7 in the lag time between ipsilateral and contralateral paw for times to contact or remove the label (one-way ANOVA with Bonferroni’s posttests for multiple comparisons). A trend for improved ability to contact the label in the contralateral paw was observed at day 3 in groups receiving O-PFC. Likewise, there was a trend towards improved ability to remove the label using the contralateral paw observed in all treatment groups vs normoxic control at day 7. As expected, there was significant improvement observed in the lag time to contact in all groups between day 3 and day 7 (paired *t* test). However, this improvement from day 3 to day 7 was not observed in the normoxic control group for the lag time to remove the label, while it was observed in the three treatment groups (paired *t* test).

### In Vivo Study 3—Efficacy of O-PFC in a Model of Permanent MCAO

**Fig. 6:**
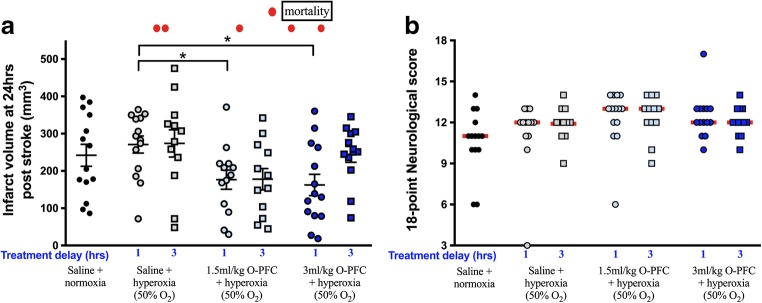
Study 3. **a** Infarct size measured from T2 MRI scans at 24 h poststroke in rats exposed to permanent MCAO. Data are presented as mean ± SD (*n* = 14 per group). *p < 0.05, 1.5 or 3 ml/kg O-PFC + hyperoxia vs saline + hyperoxia alone (50% O_2_), started 1 h poststroke. One-way ANOVA with Bonferroni’s multiple comparison test. **b** Functional outcome assessed with the 18-point neurological score. High score = improved function, red bars indicate median

O-PFC + 50% O_2_ (1.5 and 3 ml/kg, iv) starting 1 h poststroke reduced infarct size by 27% and 33%, respectively, compared to normoxia controls (Fig. 6a). This effect did not reach statistical significance compared to the normoxia group but was significantly smaller than the time-matched hyperoxia group (Fig. 6a). The trend towards efficacy was reduced when treatment was delayed until 3 h poststroke, in the 3 ml/kg O-PFC group. In the 3-h treatment, delayed animals receiving 1.5 ml/kg O-PFC + 50% O_2_ a non-significant 35% reduction in infarct size was observed, compared to the time-matched hyperoxia-alone group (Fig. 6a).

A 1–2 point improvement in the 18-point neurological score in both the 1.5 ml/kg and 3 ml/kg O-PFC + 50% O_2_ groups compared to normoxic controls did not achieve statistical significance (Fig. 6b).

**Fig. 7:**
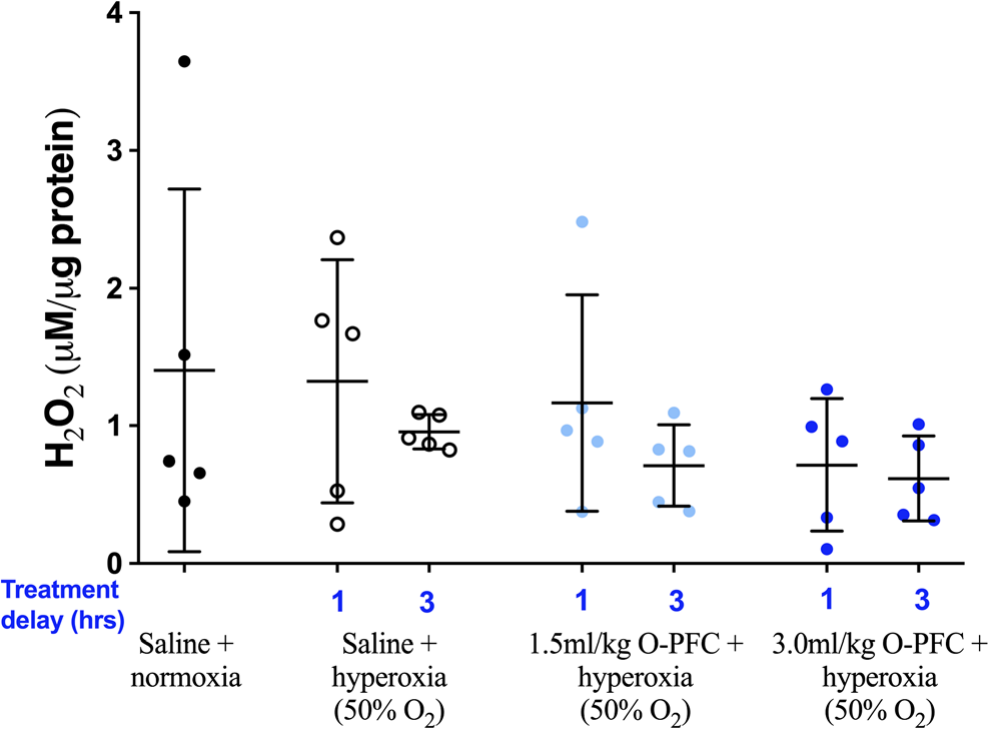
Study 3. H_2_O_2_ activity at 24 h after pMCAO, in tissue samples from the ischaemic cortex of randomly selected animals. Data presented as mean ± SD (*n* = 5 per group)

There was no evidence for increased oxidative stress levels in cortical samples from the ischaemic hemisphere 24 h after stroke in the hyperoxia-alone or O-PFC + 50% O_2_ groups when compared to the control normoxia group (Fig. 7).

### In Vivo Study 4—Investigation of Potential Interactions Between O-PFC + Hyperoxia and rt-PA in an Embolic Stroke Model

**Fig. 8:**
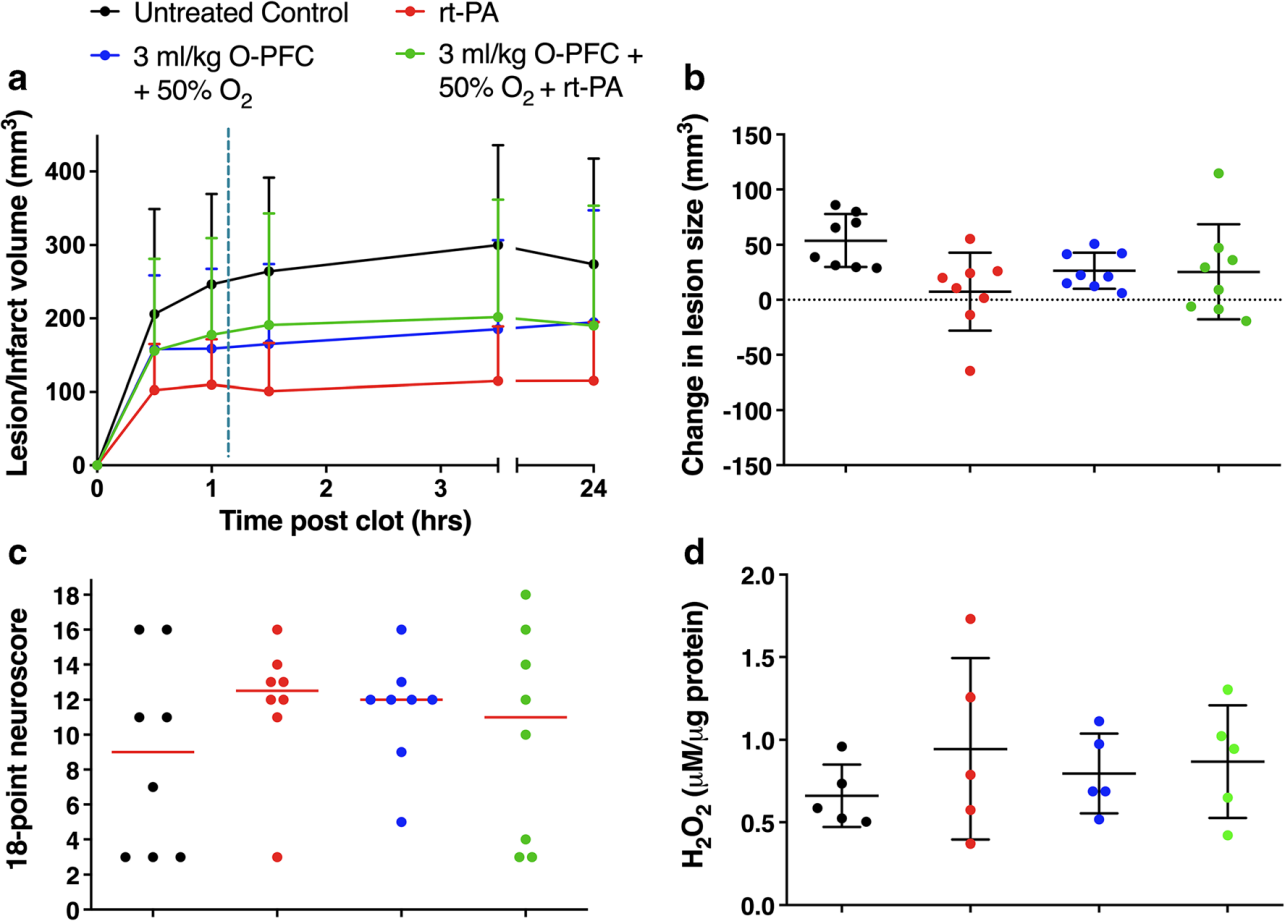
Study 4. **a** Graph showing initial lesion size and lesion growth during the acute MRI scanning protocol in the embolic stroke model. Animals were re-scanned using T_2_-weighted imaging for infarct size at 24 h. Rats were treated with rt-PA (0.9 mg/ml) or 3 ml/kg O-PFC + 50% O_2_ ± rt-PA. Treatment was initiated after the first two DWI scans approximately 75 min after stroke onset (dashed vertical line) Data are presented as mean ± SD (*n* = 8 per group). **b** Absolute change in lesion size for each group measured from pretreatment baseline (1 h poststroke) to end of acute scanning protocol (3.5 h poststroke). Data presented as mean ± SD. **c** Eighteen-point neurological score (higher score = improved function, red bar representing median). Animals not surviving to 24 h were assigned a score of 3. **d** H_2_O_2_ activity at 24 h poststroke, in tissue samples from the ischaemic cortex of randomly selected animals. Data presented as mean ± SD

Variability in the size and location of the infarct in rodent embolic stroke models is inherently greater than in filament-induced permanent and transient MCAO models. By chance, baseline (30 min and 1 h poststroke) DWI lesions, prior to treatment, were smallest in the rt-PA group (mean lesion size = 102.5 ± 62.3 mm^3^) and largest in the control normoxia group (mean lesion size = 205.9 ± 142.8 mm^3^) (Fig. 8a). Consequently, acute growth in the ischaemic lesion, from baseline (1 h poststroke) to 3.5 h poststroke, was used to assess any influence of O-PFC + hyperoxia or interaction with rt-PA. The greatest increase in lesion size was in the untreated control group, but there were no statistically significant differences in acute lesion growth between groups (Fig. 8b). Therefore, coadministration of O-PFC + 50% O_2_ with rt-PA did not display any negative effects with respect to lesion growth in this embolic stroke study.

There was a suggestion towards an improvement in the 18-point (non-significant 2–3 point increase) neurological score in all treatment groups compared to the normoxic control group (Fig. 8c).

Ischaemic cortex samples, taken 24 h after embolic stroke, revealed no significant differences in oxidative stress levels between the treatment groups (Fig. 8d). The highest levels of H_2_O_2_ were recorded in the rt-PA group.

## Discussion

Early recanalisation of occluded cerebral arteries by thrombolysis and/or thrombectomy, significantly increases the likelihood of independence after stroke [37]. Reperfusion, and thereby rescue, of the penumbra is the assumed mechanism. Potentially salvageable penumbral tissue is hypoperfused and cells switch to anaerobic metabolism in the absence of oxygen, which thereby significantly reduces their capacity to generate sufficient ATP for normal function, and consequently limits survival within the penumbra [38]. Recanalisation of medium and large intracranial arteries visualised by conventional angiographic imaging methods does not address microvascular tissue perfusion. Rat brain capillaries have an average diameters of about 2.9 μm [39], and rat RBCs have a diameter of about 6.5 μm. To pass through these tiny blood vessels, RBCs require to rearrange their cytoskeleton, which is made difficult by stiffening consequent to acidosis in ischaemic tissues. O-PFC micelles however are 1/30th the size of rat RBCs (diameter of about 200 nm) and are able to penetrate the smallest blood vessels much more readily than RBCs, enabling oxygen delivery to ischaemic tissues.

During the development of GOLD imaging for diagnostic penumbral detection, we uncovered a serendipitous benefit, which was support of penumbral tissue, presumably through the restoration of oxygen levels (Figs. 5 and 6 in [14]). Thus, O-PFC combined with 50% normobaric hyperoxia (GOLD therapy) could support the penumbra when administered prior to reperfusion. This adjunctive therapy could potentially extend the time windows for rt-PA and thrombectomy, allowing more patients to be treated. Further, this therapy could potentially be of benefit when early recanalisation is not achieved, by improving oxygen delivery to penumbra via collateral flow. We have therefore explored the potential benefits of maintained normobaric hyperoxia using PFC emulsion O-PFC and 50% O_2_ in both permanent and transient MCAO rodent stroke models and investigated the possibility of any adverse interactions with rt-PA in an embolic stroke model.

Overall, the in vivo studies show evidence of efficacy in infarct size and neurological scores for poststroke treatment with O-PFC + 50% O_2_ in clinically relevant models. O-PFC + 50% O_2_ appears more effective than hyperoxia alone (studies 1, 2 and 3) with a proposed optimum dosing regime of 3 ml/kg + 50% O_2_ delivered within 1 h of stroke. This regime induced a significant 45% reduction in infarct size and a 2-point improvement in neurological score in the tMCAO model, which induces a surge reperfusion similar to thrombectomy. To our knowledge, O-PFC + 50% O_2_ has not previously been tested in a t-MCAO model. Delayed (3 h) administration in p-MCAO (study 3) and a higher dose of 6 ml/kg in t-MCAO (studies 1 and 2) were not effective. Infarct size and behavioural outcome are inherently variable in rodent stroke models, despite standardisation of techniques (e.g. maintaining consistency in the induction of stroke and control of physiological variables under anaesthesia). Perhaps this is the reason that O-PFC + 50% O_2_ did not consistently achieve statistical significance compared to the control group (normoxia + vehicle). In study 3 (pMCAO), the 1.5 and 3 ml/kg (administered 1 h poststroke) O-PFC + 50% O_2_ group infarcts were not significantly smaller than the control normoxia group, although there was a trend towards smaller infarcts in both O-PFC treated groups (1.5 ml/kg O-PFC decreased by 27%, *p* = 0.10, and 3.0 ml/kg O-PFC decreased by 33%, *p* = 0.06, when compared to normoxia + vehicle). However, infarct size was significantly smaller in both O-PFC-treated groups when compared to the hyperoxia + saline group, which had larger infarcts and a smaller SD than the control normoxia group. It was notable that there was a loss of the efficacy observed when administering O-PFC at 1 h, when delaying treatment until 3 h poststroke in the permanent MCAO model (study 3). The loss of efficacy is not surprising as penumbra would be expected to decrease during the hours following MCAO a model with no reperfusion. However, there was still a trend towards smaller infarcts in the group receiving the lower dose of O-PFC administered 3 h post-MCAO. We have previously shown evidence for existence of penumbra up to 3 h post-MCAO in this model using advanced imaging techniques [14]. These data are encouraging, but the true therapeutic potential can only be realised through clinical testing. At present, there is a very limited literature for PFC studies in stroke models for comparison. One published study in rat p-MCAO reported a significant 12% reduction in brain damage using a very early endpoint after stroke (8 h) with O-PFC (1 ml/100 g, i.v.) + 100% normobaric O_2_ administered immediately after induction of stroke [26] while the same study design replacing normobaric with hyperbaric oxygenation was ineffective [40]. Alternative PFC emulsions (e.g. dodecafluoropentane (DDFP), 0.6 ml/kg i.v.) have been tested in rodent and rabbit stroke models under normoxic conditions. In rat p-MCAO, infarct size and neurological status were improved within 6 h of stroke after single (1 h poststroke) and repeated DDFP administration [41] and this regime was similarly effective in a rabbit embolic stroke model [42–44]. Encouraging for the field of PFC research in stroke, DDFP is currently being investigated in an ongoing phase 1b clinical trial in acute ischaemic stroke patients (NCT02963376; https://clinicaltrials.gov).

An adverse interaction with rt-PA would significantly limit the development of any new acute stroke therapy. Study 4, conducted to investigate this point, did not uncover any significant increase in damage, neurological deficit or incidence of haemorrhage when rt-PA was combined with O-PFC + 50% hyperoxia in an embolic stroke model. This study was not powered to detect efficacy, but a rabbit embolic stroke model combining rt-PA and the PFC DDFP reported reduced infarct size and improved neurological scores for the combination compared to rt-PA alone [44].

Delivering oxygen to injured hypoperfused tissues might raise concerns about the potential to exacerbate oxidative stress and reperfusion-related injuries [45]. However, a recent review on normobaric hyperoxia treatment in acute ischaemic stroke reported no evidence for increase reactive oxygen species or oxidative stress when normobaric hyperoxia is applied for a short duration (up to 8 h following MCAO): the benefits appear to significantly outweigh the risks of potential increases in reactive oxygen species [46]. We found no evidence for increased levels of oxidative stress in rat models of t-MCAO treated with O-PFC + 50% O_2_. No negative interactions on lesion growth, infarct size or neurological deficit were identified when O-PFC + 50% O_2_ was combined with rt-PA in an embolic stroke model. Furthermore, in vitro experiments and data from blood and brain tissue samples showed no evidence of increased oxidative stress following administration of O-PFC + hyperoxia. These data suggest that O-PFC + 50% O_2_ does not put injured ischaemic tissue at increased risk of reperfusion or oxidative stress-related injuries.

Clinical stroke imaging studies using various methods indicate survival of penumbral tissue beyond the current 4.5-h treatment window for rt-PA [47], and recent results from endovascular (DAWN and DEFUSE-3) and intravenous thrombolytic (EXTEND) trials support the efficacy of reperfusion therapy in late time windows based on imaging signatures. The rate of progression of tissue damage varies markedly among individuals [10], leading to the concept of “fast” and “slow” progressors. In addition to acting as a contrast agent to image the penumbra, the O-PFC + 50% O_2_ approach has the added and unique advantage of potentially improving penumbral survival. The observations of possibly smaller infarct volumes among O-PFC-treated rats compared to control after permanent MCAO in particular might have relevance to the “slow progressor” group in a clinical setting.

Despite the best efforts to power the main efficacy studies (studies 2 and 3) to detect statistical differences in the key endpoints, it is likely they remained underpowered due to the known variability associated with preclinical stroke models. A further potential limitation relates to a reliance on neurological scores and one sensorimotor behavioural test to assess functional outcome. Given the subjective nature of neurological scoring, it is possible that studies could have been improved by assessing a greater range of sensorimotor tests with longer end-points, although there remains a lack of consensus on the appropriate tests to be used with a large variation in behavioural tests being used across different preclinical stroke models. All in vivo studies were performed under general anaesthesia which can influence physiological variables including blood pressure, respiration, blood gases and body temperature. Anaesthesia is required for stroke surgery and in vivo imaging in rodent models. However, the confounding effect of general anaesthesia on the results obtained was minimised by monitoring physiological variables throughout the experiments and maintaining these within physiological limits.

In summary, O-PFC with 50% O_2_ hyperoxia delivered as constituents of GOLD penumbral imaging has the potential to improve stroke outcome and be used as an adjunct for reperfusion therapy. The translational potential of the unique, dual diagnostic and therapeutic (theranostic) properties of this technology will be investigated in clinical trials in acute stroke patients scheduled to begin during the second half of 2018.
